# 5,11,17,23-Tetra-*tert*-butyl-25,27-bis­[2-(4-nitro­phen­oxy)eth­oxy]calix[4]arene-26,28-diol acetonitrile tetra­solvate

**DOI:** 10.1107/S1600536809018212

**Published:** 2009-05-20

**Authors:** Jiu-Mao Yuan, Yong-Hong Gao, Jian-Ping Ma, Dian-Shun Guo

**Affiliations:** a Department of Chemistry, Shandong Normal University, Jinan 250014, People’s Republic of China

## Abstract

In the crystal structure of the title compound, C_60_H_70_N_2_O_10_·4CH_3_CN, the calix[4]arene mol­ecule adopts an open-cone conformation with two intra­molecular O—H⋯O hydrogen bonds. The four benzene rings of the calix[4]arene are twisted to the mean plane defined by four methyl­ene C atoms bridging the benzene rings, with dihedral angles ranging from 57.74 (10) to 65.99 (12)°. Two pendant nitro­phenyl rings are nearly perpendicular to each other, the dihedral angle being 70.9 (3)°. The asymmetric unit of the crystal structure contains four acetonitrile solvent mol­ecules, one of which lies in the calix cavity and makes C—H⋯π inter­actions and another links with the calix[4]arene *via* C—H⋯O hydrogen bonding. One *tert*-butyl group is disordered over two sets of sites, with a 0.736 (13):0.264 (13) occupancy ratio.

## Related literature

For general background to the chemistry of calix[4]arenes, see: Gutsche (1998 ▶). For related crystal structures, see: Singh *et al.* (2004 ▶); Bolte *et al.* (2003 ▶); Zeng *et al.* (2002 ▶); Gale *et al.* (1998 ▶); Drew *et al.* (1997 ▶); Böhmer *et al.* (1993 ▶); Bugge *et al.* (1992 ▶). For C—H⋯π contacts, see: Tsuzuki *et al.* (2000 ▶); Umezawa *et al.* (1998 ▶). For inclusion complexes, see: McKervey *et al.* (1986 ▶).
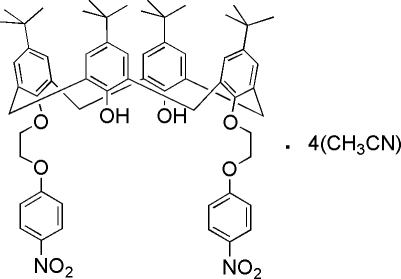


## Experimental

### 

#### Crystal data

C_60_H_70_N_2_O_10_·4C_2_H_3_N
*M* *_r_* = 1143.40
Triclinic, 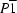

*a* = 13.195 (3) Å
*b* = 13.388 (3) Å
*c* = 19.692 (5) Å
α = 71.978 (3)°
β = 84.022 (3)°
γ = 82.230 (3)°
*V* = 3270.5 (13) Å^3^
*Z* = 2
Mo *K*α radiation
μ = 0.08 mm^−1^
*T* = 173 K
0.51 × 0.35 × 0.15 mm

#### Data collection

Bruker SMART CCD area-detector diffractometer
Absorption correction: none
15816 measured reflections
11155 independent reflections
7114 reflections with *I* > 2σ(*I*)
*R*_int_ = 0.049

#### Refinement

*R*[*F* ^2^ > 2σ(*F* ^2^)] = 0.114
*wR*(*F* ^2^) = 0.349
*S* = 1.05
11155 reflections
775 parameters
5 restraints
H-atom parameters constrained
Δρ_max_ = 0.78 e Å^−3^
Δρ_min_ = −0.64 e Å^−3^

### 

Data collection: *SMART* (Bruker, 1999 ▶); cell refinement: *SAINT* (Bruker, 1999 ▶); data reduction: *SAINT*; program(s) used to solve structure: *SHELXTL* (Sheldrick, 2008 ▶); program(s) used to refine structure: *SHELXTL*; molecular graphics: *SHELXTL*; software used to prepare material for publication: *SHELXTL*.

## Supplementary Material

Crystal structure: contains datablocks I, global. DOI: 10.1107/S1600536809018212/xu2512sup1.cif

Structure factors: contains datablocks I. DOI: 10.1107/S1600536809018212/xu2512Isup2.hkl

Additional supplementary materials:  crystallographic information; 3D view; checkCIF report

## Figures and Tables

**Table 1:** Hydrogen-bond geometry (Å, °)

*D*—H⋯*A*	*D*—H	H⋯*A*	*D*⋯*A*	*D*—H⋯*A*
O1—H1⋯O3	0.82	1.99	2.802 (6)	171
O2—H2⋯O4	0.82	1.97	2.781 (4)	176
C50—H50*C*⋯O7^i^	0.96	2.59	3.487 (10)	156
C68—H68*B*⋯O10	0.96	2.39	3.247 (13)	148
C65—H65*A*⋯*Cg*1	0.96	2.66	3.590 (6)	163

Symmetry code: (i) 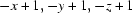. *Cg*1 is the centroid of the C42–C47 ring.

**Fig. 1.**
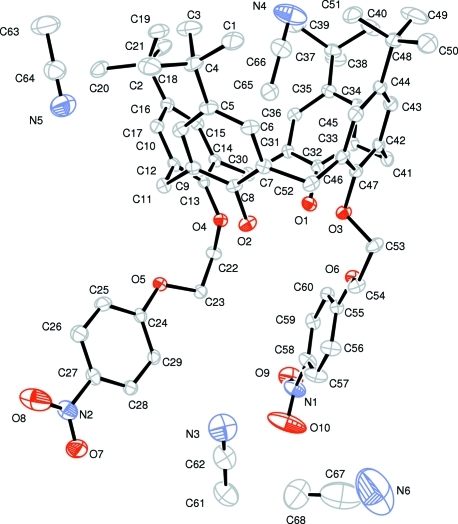
The molecular structure of the title compound, with displacement ellipsoids drawn at the 30% probability level. The minor disordered component and H atoms have been omitted for clarity.

## supplementary crystallographic information

### Comment

Calix[4]arenes have been attracting much interest because they possess a versatile three-dimensional cavity and are ideal scaffolds for the construction of supramolecular systems. In particular, the lower or upper rim of a calix[4]arene platform can be modified to achieve more sophisticated receptors with a specific affinity and selectivity for ion recognition (Gutsche, 1998). Several crystal structures of 1,3-substituted cone calix[4]arene derivatives (Singh *et al.*, 2004; Bolte *et al.*, 2003; Zeng *et al.*, 2002; Gale *et al.*, 1998; Drew *et al.*, 1997; Böhmer *et al.*, 1993; Bugge *et al.*, 1992) have been described. We report here the crystal structure of a new 1,3-substituted calix[4]arene, C_60_H_70_N_2_O_10_.4CH_3_CN, namely 5,11,17,23-tetra-*tert*-butyl-25,27-bis[2-(4-nitrophenoxy)ethoxy]-26,28-dihydroxycalix[4]arene acetonitrile tetrasolvate.

In the crystal structure of the title compound, as shown in Fig. 1, the molecule of the calix[4]arene adopts an open-cone conformation, in which either phenol hydroxy group links with one neighboring ethereal O atom *via* an intramolecular O—H···O hydrogen bond (Table 1) and one *t*-butyl group shows rotational disorder. The four benzene rings of the calix[4]arene are twisted to the virtual plane defined by four methylene C atoms bridging the phenolic rings with dihedral angles ranging from 57.74 (10) to 65.99 (12)°. Two pendant nitrophenyl rings are nearly perpendicular to each other, the dihedral angle being 70.9 (3)°. This conformation results in a distance of 4.005 (6) Å between diametrically opposed atoms O1 and O2, almost same as 4.289 (6) Å between O3 and O4.

There are four acetonitrile solvate molecules in the asymmetric unit of the crystal structure, one of which lies in the calix cavity with C—H···*π* contacts (Umezawa *et al.*, 1998; Tsuzuki *et al.*, 2000) and another links with the calix[4]arene *via* C—H···O hydrogen bonding (Table 1). The apolar end of the acetonitrile is held in the cavity, while the polar one remains outside, similar to the related cone calix[4]arene system (McKervey *et al.*, 1986) where an acetonitrile molecule is included. The intermolecular C—H···O hydrogen bonds (Table 1) and the remaining acetonitrile molecules stabilize the molecular packing.

### Experimental

To a refluxing suspension of *p*-*tert*-butylcalix[4]arene (1.112 g, 1.50 mmol) and anhydrous potassium carbonate (0.228 g, 1.65 mmol) in dry acetonitrile (15 ml) was added 2-(4-nitrophenoxy)ethyl-4-methylbenzenesulfonate (1.144 g, 3.00 mmol) in dry acetonitrile (15 ml) dropwise. The mixture was stirred and refluxed under a nitrogen atmosphere for 46 h and cooled to room temperature. The solvent was removed under reduced pressure. The residue was neutralized with diluted hydrochloric acid and extracted with dichloromethane. The organic layer was washed with saturated sodium hydrogen carbonate and brine, and dried over anhydrous magnesium sulfate. Removal of the solvent under reduced pressure, the residue was purified by flash column chromatography (silica gel, ethyl acetate/hexane/dichloromethane = 1:12:4, *R*_F_ = 1/2) to give the title compound in 88% yield as a white solid, m.p. 397–399 K. Single crystals suitable for X-ray diffraction analysis were obtained from slow evaporation of a solution in acetonitrile at 298 K.

### Refinement

All H atoms were placed in geometrically idealized positions and refined using a riding model, with C—H distances of 0.93–0.97 Å, and with *U*_iso_(H) values of 1.5*U*_eq_(C) for methyl H atoms and 1.2*U*_eq_(C) for the other H atoms. In the title compound, one *tert*-butyl group (C1–C4) is rotational disordered over two sites; the site-occupancies were refined to 0.736 (13):0.264 (13). The C—C bond lengths involving the disordered atoms were restrained to be similar. The C67—C68 and C67—N6 bond lengths were restrained. As the quality of the crystal is poor the accuracy of the determination is low.

### Crystal data

**Table:**

C_60_H_70_N_2_O_10_·4C_2_H_3_N	*Z* = 2
*M**_r_* = 1143.40	*F*(000) = 1224
Triclinic, *P*1	*D*_x_ = 1.161 Mg m^−^^3^
Hall symbol: -P 1	Mo *K*α radiation, λ = 0.71073 Å
*a* = 13.195 (3) Å	Cell parameters from 4717 reflections
*b* = 13.388 (3) Å	θ = 2.6–27.2°
*c* = 19.692 (5) Å	µ = 0.08 mm^−^^1^
α = 71.978 (3)°	*T* = 173 K
β = 84.022 (3)°	Block, colourless
γ = 82.230 (3)°	0.51 × 0.35 × 0.15 mm
*V* = 3270.5 (13) Å^3^	

### Data collection

**Table:**

Bruker SMART CCD area-detector diffractometer	7114 reflections with *I* > 2σ(*I*)
Radiation source: fine-focus sealed tube	*R*_int_ = 0.049
graphite	θ_max_ = 25.0°, θ_min_ = 1.6°
φ and ω scans	*h* = −15→15
15816 measured reflections	*k* = −15→15
11155 independent reflections	*l* = −13→23

### Refinement

**Table:**

Refinement on *F*^2^	Primary atom site location: structure-invariant direct methods
Least-squares matrix: full	Secondary atom site location: difference Fourier map
*R*[*F*^2^ > 2σ(*F*^2^)] = 0.114	Hydrogen site location: inferred from neighbouring sites
*wR*(*F*^2^) = 0.349	H-atom parameters constrained
*S* = 1.05	*w* = 1/[σ^2^(*F*_o_^2^) + (0.1473*P*)^2^ + 8.6696*P*] where *P* = (*F*_o_^2^ + 2*F*_c_^2^)/3
11155 reflections	(Δ/σ)_max_ = 0.005
775 parameters	Δρ_max_ = 0.78 e Å^−^^3^
5 restraints	Δρ_min_ = −0.64 e Å^−^^3^

### Special details

**Table:**

Experimental. ^1^H NMR (300 MHz, CDCl_3_): *δ* 8.19 (d, 4H, *J* = 9.20 Hz), 7.09 (s, 2H), 7.06 (s, 4H), 6.99 (d, 4H, *J* = 9.20 Hz), 6.82 (s, 4H), 4.35 (s, 8H), 4.33 (d, 4H, *J* = 13.02 Hz), 3.31 (d, 4H, *J* = 13.02 Hz), 1.29 (s, 18H), 0.98 (s, 18H).
Geometry. All e.s.d.'s (except the e.s.d. in the dihedral angle between two l.s. planes) are estimated using the full covariance matrix. The cell e.s.d.'s are taken into account individually in the estimation of e.s.d.'s in distances, angles and torsion angles; correlations between e.s.d.'s in cell parameters are only used when they are defined by crystal symmetry. An approximate (isotropic) treatment of cell e.s.d.'s is used for estimating e.s.d.'s involving l.s. planes.
Refinement. Refinement of *F*^2^ against ALL reflections. The weighted *R*-factor *wR* and goodness of fit *S* are based on *F*^2^, conventional *R*-factors *R* are based on *F*, with *F* set to zero for negative *F*^2^. The threshold expression of *F*^2^ > σ(*F*^2^) is used only for calculating *R*-factors(gt) *etc*. and is not relevant to the choice of reflections for refinement. *R*-factors based on *F*^2^ are statistically about twice as large as those based on *F*, and *R*- factors based on ALL data will be even larger.

### Fractional atomic coordinates and isotropic or equivalent isotropic displacement parameters (Å^2^)

**Table:**

	*x*	*y*	*z*	*U*_iso_*/*U*_eq_	Occ. (<1)
C1	0.2547 (7)	0.3466 (10)	0.8947 (5)	0.069 (3)	0.739 (13)
H1A	0.2256	0.3367	0.9428	0.104*	0.739 (13)
H1B	0.2453	0.2872	0.8795	0.104*	0.739 (13)
H1C	0.2213	0.4099	0.8635	0.104*	0.739 (13)
C2	0.3929 (11)	0.4421 (10)	0.9200 (6)	0.072 (4)	0.739 (13)
H2A	0.4656	0.4430	0.9201	0.107*	0.739 (13)
H2B	0.3626	0.4301	0.9679	0.107*	0.739 (13)
H2C	0.3636	0.5088	0.8901	0.107*	0.739 (13)
C3	0.4147 (10)	0.2469 (7)	0.9405 (5)	0.072 (4)	0.739 (13)
H3A	0.4877	0.2435	0.9416	0.107*	0.739 (13)
H3B	0.3988	0.1930	0.9217	0.107*	0.739 (13)
H3C	0.3834	0.2361	0.9881	0.107*	0.739 (13)
C4	0.3731 (5)	0.3569 (5)	0.8920 (3)	0.0485 (15)	
C5	0.4236 (4)	0.3688 (4)	0.8157 (3)	0.0390 (13)	
C6	0.3685 (4)	0.3745 (4)	0.7580 (3)	0.0370 (12)	
H6	0.2978	0.3740	0.7649	0.044*	
C7	0.4149 (4)	0.3810 (4)	0.6897 (3)	0.0306 (11)	
C8	0.5219 (4)	0.3828 (4)	0.6790 (2)	0.0303 (11)	
C9	0.5803 (4)	0.3783 (4)	0.7355 (3)	0.0322 (11)	
C10	0.5309 (4)	0.3711 (4)	0.8028 (3)	0.0363 (12)	
H10	0.5698	0.3677	0.8404	0.044*	
C11	0.6975 (4)	0.3713 (4)	0.7274 (3)	0.0342 (11)	
H11A	0.7194	0.4094	0.6787	0.041*	
H11B	0.7218	0.4040	0.7594	0.041*	
C12	0.7438 (3)	0.2563 (4)	0.7448 (3)	0.0293 (11)	
C13	0.7655 (3)	0.2053 (4)	0.6906 (2)	0.0275 (10)	
C14	0.7975 (3)	0.0963 (4)	0.7087 (3)	0.0285 (10)	
C15	0.8073 (3)	0.0420 (4)	0.7807 (3)	0.0315 (11)	
H15	0.8274	−0.0303	0.7932	0.038*	
C16	0.7887 (4)	0.0896 (4)	0.8355 (3)	0.0335 (11)	
C17	0.7542 (4)	0.1987 (4)	0.8148 (3)	0.0326 (11)	
H17	0.7381	0.2324	0.8500	0.039*	
C18	0.8001 (4)	0.0302 (4)	0.9147 (3)	0.0416 (13)	
C19	0.6944 (5)	0.0345 (6)	0.9561 (3)	0.0621 (18)	
H19A	0.6659	0.1069	0.9476	0.093*	
H19B	0.7022	0.0024	1.0064	0.093*	
H19C	0.6494	−0.0029	0.9400	0.093*	
C20	0.8751 (5)	0.0844 (6)	0.9452 (3)	0.0568 (17)	
H20A	0.9420	0.0787	0.9214	0.085*	
H20B	0.8790	0.0502	0.9956	0.085*	
H20C	0.8502	0.1577	0.9371	0.085*	
C21	0.8431 (6)	−0.0850 (5)	0.9263 (3)	0.072 (2)	
H21A	0.7986	−0.1193	0.9073	0.107*	
H21B	0.8476	−0.1200	0.9766	0.107*	
H21C	0.9101	−0.0883	0.9024	0.107*	
C22	0.8396 (4)	0.2765 (4)	0.5701 (3)	0.0354 (12)	
H22A	0.8345	0.2354	0.5380	0.042*	
H22B	0.9016	0.2491	0.5953	0.042*	
C23	0.8444 (4)	0.3907 (4)	0.5278 (3)	0.0322 (11)	
H23A	0.8990	0.3969	0.4902	0.039*	
H23B	0.7802	0.4200	0.5058	0.039*	
C24	0.8708 (3)	0.5528 (4)	0.5498 (3)	0.0313 (11)	
C25	0.8717 (5)	0.6058 (5)	0.6011 (3)	0.0485 (15)	
H25	0.8677	0.5678	0.6494	0.058*	
C26	0.8784 (5)	0.7130 (5)	0.5813 (3)	0.0556 (16)	
H26	0.8787	0.7473	0.6157	0.067*	
C27	0.8846 (4)	0.7684 (4)	0.5092 (3)	0.0388 (12)	
C28	0.8843 (4)	0.7170 (4)	0.4582 (3)	0.0366 (12)	
H28	0.8900	0.7550	0.4099	0.044*	
C29	0.8755 (4)	0.6102 (4)	0.4779 (3)	0.0334 (11)	
H29	0.8727	0.5769	0.4432	0.040*	
C30	0.8120 (3)	0.0326 (4)	0.6552 (3)	0.0295 (10)	
H30A	0.8727	−0.0178	0.6652	0.035*	
H30B	0.8221	0.0799	0.6071	0.035*	
C31	0.7182 (3)	−0.0269 (4)	0.6594 (2)	0.0280 (10)	
C32	0.6346 (4)	0.0221 (4)	0.6168 (2)	0.0289 (10)	
C33	0.5466 (4)	−0.0287 (4)	0.6241 (2)	0.0307 (11)	
C34	0.5401 (4)	−0.1265 (4)	0.6752 (3)	0.0368 (12)	
H34	0.4806	−0.1592	0.6805	0.044*	
C35	0.6199 (4)	−0.1770 (4)	0.7186 (3)	0.0370 (12)	
C36	0.7094 (4)	−0.1248 (4)	0.7083 (3)	0.0324 (11)	
H36	0.7644	−0.1581	0.7358	0.039*	
C37	0.6147 (5)	−0.2837 (4)	0.7760 (3)	0.0445 (14)	
C38	0.6961 (6)	−0.3656 (5)	0.7568 (4)	0.0629 (19)	
H38A	0.6818	−0.3745	0.7123	0.094*	
H38B	0.6947	−0.4319	0.7938	0.094*	
H38C	0.7626	−0.3417	0.7522	0.094*	
C39	0.6358 (6)	−0.2736 (5)	0.8489 (3)	0.0618 (18)	
H39A	0.7029	−0.2519	0.8461	0.093*	
H39B	0.6321	−0.3406	0.8849	0.093*	
H39C	0.5855	−0.2219	0.8611	0.093*	
C40	0.5086 (5)	−0.3241 (6)	0.7832 (4)	0.068 (2)	
H40A	0.4562	−0.2715	0.7918	0.103*	
H40B	0.5065	−0.3879	0.8225	0.103*	
H40C	0.4971	−0.3382	0.7399	0.103*	
C41	0.4530 (4)	0.0235 (4)	0.5799 (3)	0.0356 (12)	
H41A	0.4764	0.0657	0.5327	0.043*	
H41B	0.4163	−0.0311	0.5741	0.043*	
C42	0.3802 (3)	0.0938 (4)	0.6162 (3)	0.0323 (11)	
C43	0.3087 (4)	0.0466 (4)	0.6706 (3)	0.0380 (12)	
H43	0.3024	−0.0250	0.6812	0.046*	
C44	0.2465 (4)	0.1071 (4)	0.7093 (3)	0.0363 (12)	
C45	0.2605 (4)	0.2121 (4)	0.6934 (3)	0.0374 (12)	
H45	0.2208	0.2516	0.7198	0.045*	
C46	0.3309 (3)	0.2633 (4)	0.6397 (2)	0.0333 (11)	
C47	0.3896 (3)	0.2003 (4)	0.6010 (2)	0.0319 (11)	
C48	0.1656 (4)	0.0584 (5)	0.7686 (3)	0.0496 (15)	
C49	0.1701 (7)	−0.0603 (6)	0.7842 (5)	0.092 (3)	
H49A	0.1507	−0.0758	0.7432	0.138*	
H49B	0.1237	−0.0880	0.8246	0.138*	
H49C	0.2385	−0.0922	0.7946	0.138*	
C50	0.0580 (5)	0.1101 (7)	0.7444 (4)	0.079 (2)	
H50A	0.0553	0.1855	0.7333	0.118*	
H50B	0.0073	0.0845	0.7823	0.118*	
H50C	0.0446	0.0925	0.7028	0.118*	
C51	0.1810 (5)	0.0845 (6)	0.8371 (3)	0.065 (2)	
H51A	0.2480	0.0550	0.8529	0.098*	
H51B	0.1301	0.0550	0.8737	0.098*	
H51C	0.1744	0.1597	0.8277	0.098*	
C52	0.3504 (4)	0.3764 (4)	0.6306 (3)	0.0348 (12)	
H52A	0.2854	0.4196	0.6321	0.042*	
H52B	0.3859	0.4050	0.5843	0.042*	
C53	0.4231 (4)	0.2907 (5)	0.4771 (3)	0.0424 (13)	
H53A	0.3676	0.3462	0.4771	0.051*	
H53B	0.3975	0.2360	0.4630	0.051*	
C54	0.5112 (4)	0.3343 (4)	0.4267 (3)	0.0385 (12)	
H54A	0.4889	0.3668	0.3787	0.046*	
H54B	0.5384	0.3871	0.4420	0.046*	
C55	0.6794 (4)	0.2639 (4)	0.3893 (3)	0.0308 (11)	
C56	0.6960 (4)	0.3571 (4)	0.3360 (3)	0.0470 (15)	
H56	0.6447	0.4135	0.3263	0.056*	
C57	0.7906 (5)	0.3650 (5)	0.2974 (4)	0.0610 (19)	
H57	0.8027	0.4267	0.2611	0.073*	
C58	0.8660 (4)	0.2816 (4)	0.3128 (3)	0.0456 (14)	
C59	0.8509 (4)	0.1895 (4)	0.3659 (3)	0.0406 (13)	
H59	0.9027	0.1336	0.3756	0.049*	
C60	0.7566 (4)	0.1811 (4)	0.4049 (3)	0.0350 (11)	
H60	0.7456	0.1196	0.4417	0.042*	
C61	1.0401 (8)	0.6203 (8)	0.1833 (6)	0.105 (3)	
H61A	1.0938	0.5636	0.1852	0.157*	
H61B	1.0696	0.6843	0.1777	0.157*	
H61C	0.9998	0.6295	0.1435	0.157*	
C62	0.9756 (6)	0.5953 (7)	0.2489 (6)	0.085 (3)	
C63	0.7033 (10)	0.2776 (8)	1.0540 (4)	0.115 (4)	
H63A	0.7504	0.2147	1.0597	0.173*	
H63B	0.6344	0.2588	1.0645	0.173*	
H63C	0.7179	0.3141	1.0862	0.173*	
C64	0.7141 (6)	0.3450 (6)	0.9816 (4)	0.072 (2)	
C65	0.5154 (4)	0.0915 (5)	0.7801 (3)	0.0500 (15)	
H65A	0.4639	0.1254	0.7469	0.075*	
H65B	0.5748	0.0659	0.7553	0.075*	
H65C	0.5338	0.1413	0.8015	0.075*	
C66	0.4757 (5)	0.0040 (5)	0.8351 (4)	0.0561 (17)	
C67	1.0242 (10)	0.3631 (11)	0.0428 (9)	0.191 (8)	
C68	1.1128 (8)	0.3882 (10)	0.0768 (6)	0.124 (4)	
H68A	1.1753	0.3493	0.0652	0.186*	
H68B	1.0995	0.3686	0.1278	0.186*	
H68C	1.1194	0.4625	0.0587	0.186*	
C1'	0.443 (2)	0.382 (3)	0.9414 (12)	0.080 (11)	0.261 (13)
H1'1	0.4054	0.3841	0.9854	0.120*	0.261 (13)
H1'2	0.4680	0.4496	0.9180	0.120*	0.261 (13)
H1'3	0.5007	0.3285	0.9515	0.120*	0.261 (13)
C2'	0.321 (3)	0.2532 (16)	0.9184 (16)	0.098 (14)	0.261 (13)
H2'1	0.2713	0.2549	0.8857	0.147*	0.261 (13)
H2'2	0.2878	0.2466	0.9651	0.147*	0.261 (13)
H2'3	0.3721	0.1940	0.9208	0.147*	0.261 (13)
C3'	0.2903 (18)	0.4530 (16)	0.8839 (13)	0.067 (9)	0.261 (13)
H3'1	0.3229	0.5170	0.8691	0.101*	0.261 (13)
H3'2	0.2522	0.4472	0.9289	0.101*	0.261 (13)
H3'3	0.2448	0.4545	0.8486	0.101*	0.261 (13)
N1	0.9639 (4)	0.2891 (4)	0.2696 (3)	0.0644 (16)	
N2	0.8937 (4)	0.8818 (4)	0.4872 (3)	0.0566 (14)	
N3	0.9248 (6)	0.5756 (7)	0.3004 (5)	0.114 (3)	
N4	0.4436 (6)	−0.0641 (6)	0.8780 (5)	0.111 (3)	
N5	0.7228 (7)	0.3959 (6)	0.9247 (4)	0.100 (2)	
N6	0.9436 (18)	0.342 (2)	0.031 (2)	0.45 (3)	
O1	0.6488 (2)	0.1190 (3)	0.56909 (18)	0.0364 (8)	
H1	0.5930	0.1536	0.5602	0.055*	
O2	0.5649 (3)	0.3939 (3)	0.61067 (17)	0.0381 (9)	
H2	0.6211	0.3589	0.6125	0.057*	
O3	0.4624 (2)	0.2478 (3)	0.54692 (16)	0.0337 (8)	
O4	0.7515 (2)	0.2675 (2)	0.62072 (16)	0.0291 (7)	
O5	0.8632 (3)	0.4473 (3)	0.57552 (17)	0.0365 (8)	
O6	0.5876 (2)	0.2470 (3)	0.42839 (18)	0.0350 (8)	
O7	0.9145 (6)	0.9262 (4)	0.4246 (3)	0.0969 (15)	
O8	0.8850 (6)	0.9283 (4)	0.5324 (3)	0.0969 (15)	
O9	1.0274 (4)	0.2139 (5)	0.2778 (4)	0.1034 (16)	
O10	0.9803 (4)	0.3736 (5)	0.2274 (4)	0.1034 (16)	

### Atomic displacement parameters (Å^2^)

**Table:**

	*U*^11^	*U*^22^	*U*^33^	*U*^12^	*U*^13^	*U*^23^
C1	0.061 (6)	0.108 (9)	0.046 (5)	−0.026 (6)	0.023 (4)	−0.034 (5)
C2	0.102 (10)	0.069 (7)	0.051 (6)	−0.028 (7)	0.031 (6)	−0.031 (6)
C3	0.110 (10)	0.053 (6)	0.042 (5)	−0.007 (5)	0.022 (5)	−0.008 (4)
C4	0.054 (4)	0.062 (4)	0.031 (3)	−0.003 (3)	0.009 (2)	−0.022 (3)
C5	0.044 (3)	0.034 (3)	0.034 (3)	0.004 (2)	0.006 (2)	−0.010 (2)
C6	0.031 (3)	0.037 (3)	0.036 (3)	0.006 (2)	0.003 (2)	−0.007 (2)
C7	0.026 (2)	0.025 (2)	0.037 (3)	0.0058 (19)	−0.002 (2)	−0.008 (2)
C8	0.034 (3)	0.025 (2)	0.027 (2)	0.007 (2)	0.000 (2)	−0.0053 (19)
C9	0.038 (3)	0.022 (2)	0.034 (3)	0.000 (2)	−0.001 (2)	−0.006 (2)
C10	0.045 (3)	0.031 (3)	0.033 (3)	0.003 (2)	−0.003 (2)	−0.012 (2)
C11	0.031 (3)	0.033 (3)	0.039 (3)	−0.002 (2)	−0.003 (2)	−0.012 (2)
C12	0.017 (2)	0.031 (2)	0.039 (3)	−0.0042 (19)	−0.0008 (19)	−0.010 (2)
C13	0.018 (2)	0.033 (3)	0.031 (2)	−0.0054 (19)	−0.0015 (18)	−0.008 (2)
C14	0.019 (2)	0.027 (2)	0.038 (3)	−0.0016 (18)	0.0002 (18)	−0.009 (2)
C15	0.022 (2)	0.028 (2)	0.041 (3)	0.0006 (19)	−0.003 (2)	−0.007 (2)
C16	0.025 (2)	0.037 (3)	0.038 (3)	−0.002 (2)	−0.003 (2)	−0.010 (2)
C17	0.024 (2)	0.041 (3)	0.034 (3)	−0.003 (2)	0.0000 (19)	−0.014 (2)
C18	0.036 (3)	0.049 (3)	0.036 (3)	0.004 (2)	−0.005 (2)	−0.008 (2)
C19	0.050 (4)	0.084 (5)	0.038 (3)	−0.007 (3)	0.002 (3)	−0.001 (3)
C20	0.047 (4)	0.079 (5)	0.038 (3)	0.000 (3)	−0.013 (3)	−0.008 (3)
C21	0.096 (6)	0.062 (4)	0.040 (3)	0.016 (4)	−0.016 (3)	0.002 (3)
C22	0.032 (3)	0.035 (3)	0.037 (3)	−0.003 (2)	0.005 (2)	−0.010 (2)
C23	0.033 (3)	0.033 (3)	0.032 (3)	−0.003 (2)	0.000 (2)	−0.012 (2)
C24	0.022 (2)	0.036 (3)	0.038 (3)	−0.006 (2)	0.0006 (19)	−0.014 (2)
C25	0.070 (4)	0.043 (3)	0.033 (3)	−0.014 (3)	0.003 (3)	−0.011 (2)
C26	0.077 (5)	0.054 (4)	0.044 (3)	−0.017 (3)	0.013 (3)	−0.027 (3)
C27	0.035 (3)	0.033 (3)	0.052 (3)	−0.004 (2)	0.001 (2)	−0.018 (2)
C28	0.033 (3)	0.037 (3)	0.038 (3)	−0.005 (2)	−0.008 (2)	−0.006 (2)
C29	0.029 (3)	0.040 (3)	0.035 (3)	−0.006 (2)	−0.004 (2)	−0.014 (2)
C30	0.022 (2)	0.028 (2)	0.038 (3)	0.0025 (19)	−0.0030 (19)	−0.010 (2)
C31	0.026 (2)	0.029 (2)	0.033 (2)	0.0000 (19)	0.0013 (19)	−0.017 (2)
C32	0.027 (2)	0.030 (2)	0.031 (2)	−0.0015 (19)	0.0033 (19)	−0.013 (2)
C33	0.027 (2)	0.037 (3)	0.033 (2)	−0.006 (2)	0.0027 (19)	−0.019 (2)
C34	0.034 (3)	0.038 (3)	0.044 (3)	−0.009 (2)	0.007 (2)	−0.021 (2)
C35	0.041 (3)	0.033 (3)	0.038 (3)	−0.005 (2)	0.007 (2)	−0.016 (2)
C36	0.034 (3)	0.029 (3)	0.033 (3)	0.005 (2)	−0.002 (2)	−0.012 (2)
C37	0.056 (4)	0.029 (3)	0.047 (3)	−0.007 (2)	0.006 (3)	−0.010 (2)
C38	0.080 (5)	0.033 (3)	0.068 (4)	0.000 (3)	0.012 (4)	−0.012 (3)
C39	0.092 (5)	0.046 (4)	0.042 (3)	−0.005 (3)	−0.002 (3)	−0.007 (3)
C40	0.062 (4)	0.057 (4)	0.077 (5)	−0.023 (3)	0.006 (4)	−0.003 (4)
C41	0.029 (3)	0.043 (3)	0.039 (3)	−0.007 (2)	0.000 (2)	−0.019 (2)
C42	0.019 (2)	0.046 (3)	0.033 (3)	−0.004 (2)	−0.0023 (19)	−0.013 (2)
C43	0.029 (3)	0.047 (3)	0.038 (3)	−0.006 (2)	−0.001 (2)	−0.013 (2)
C44	0.021 (2)	0.057 (3)	0.031 (3)	−0.009 (2)	0.0002 (19)	−0.012 (2)
C45	0.019 (2)	0.058 (3)	0.036 (3)	0.002 (2)	0.002 (2)	−0.018 (2)
C46	0.020 (2)	0.048 (3)	0.030 (2)	0.003 (2)	−0.0056 (19)	−0.009 (2)
C47	0.021 (2)	0.048 (3)	0.026 (2)	−0.005 (2)	0.0005 (18)	−0.011 (2)
C48	0.035 (3)	0.078 (4)	0.037 (3)	−0.019 (3)	0.008 (2)	−0.016 (3)
C49	0.105 (7)	0.084 (6)	0.087 (6)	−0.052 (5)	0.052 (5)	−0.026 (5)
C50	0.028 (3)	0.148 (8)	0.057 (4)	−0.021 (4)	0.007 (3)	−0.023 (4)
C51	0.050 (4)	0.108 (6)	0.036 (3)	−0.020 (4)	0.006 (3)	−0.017 (3)
C52	0.020 (2)	0.045 (3)	0.033 (3)	0.012 (2)	−0.0036 (19)	−0.008 (2)
C53	0.030 (3)	0.059 (4)	0.030 (3)	0.009 (2)	−0.005 (2)	−0.006 (2)
C54	0.029 (3)	0.044 (3)	0.035 (3)	0.011 (2)	−0.004 (2)	−0.007 (2)
C55	0.025 (2)	0.032 (3)	0.036 (3)	0.002 (2)	−0.0001 (19)	−0.013 (2)
C56	0.042 (3)	0.034 (3)	0.054 (3)	0.011 (2)	0.009 (3)	−0.009 (3)
C57	0.056 (4)	0.043 (3)	0.067 (4)	0.001 (3)	0.022 (3)	−0.002 (3)
C58	0.031 (3)	0.043 (3)	0.061 (4)	−0.002 (2)	0.013 (2)	−0.020 (3)
C59	0.034 (3)	0.037 (3)	0.054 (3)	0.001 (2)	−0.003 (2)	−0.020 (3)
C60	0.028 (3)	0.036 (3)	0.039 (3)	0.001 (2)	−0.004 (2)	−0.009 (2)
C61	0.084 (6)	0.100 (7)	0.130 (8)	−0.020 (5)	0.011 (6)	−0.034 (6)
C62	0.047 (4)	0.087 (6)	0.120 (8)	0.010 (4)	−0.001 (5)	−0.037 (5)
C63	0.185 (12)	0.094 (7)	0.062 (5)	−0.035 (7)	0.026 (6)	−0.020 (5)
C64	0.081 (5)	0.066 (5)	0.069 (5)	−0.011 (4)	0.007 (4)	−0.025 (4)
C65	0.035 (3)	0.059 (4)	0.054 (3)	0.001 (3)	−0.002 (3)	−0.017 (3)
C66	0.036 (3)	0.056 (4)	0.073 (4)	0.006 (3)	0.008 (3)	−0.023 (3)
C67	0.129 (12)	0.125 (11)	0.26 (2)	0.031 (10)	−0.045 (13)	0.016 (12)
C68	0.087 (7)	0.176 (11)	0.104 (8)	0.013 (7)	−0.002 (6)	−0.048 (8)
C1'	0.13 (3)	0.09 (3)	0.021 (13)	−0.01 (2)	0.019 (15)	−0.026 (16)
C2'	0.08 (2)	0.12 (3)	0.060 (18)	0.01 (2)	0.017 (17)	0.016 (18)
C3'	0.058 (17)	0.10 (2)	0.053 (15)	0.006 (15)	0.007 (12)	−0.042 (15)
N1	0.055 (3)	0.051 (3)	0.078 (4)	−0.009 (3)	0.030 (3)	−0.015 (3)
N2	0.060 (3)	0.047 (3)	0.069 (4)	−0.007 (2)	0.010 (3)	−0.031 (3)
N3	0.069 (5)	0.131 (7)	0.135 (7)	0.018 (5)	0.009 (5)	−0.045 (6)
N4	0.083 (5)	0.080 (5)	0.131 (7)	0.005 (4)	0.042 (5)	0.002 (5)
N5	0.127 (7)	0.095 (6)	0.071 (5)	−0.016 (5)	−0.011 (4)	−0.012 (4)
N6	0.32 (3)	0.28 (2)	0.72 (6)	−0.06 (2)	−0.35 (4)	0.03 (3)
O1	0.0257 (18)	0.0349 (19)	0.044 (2)	−0.0021 (14)	−0.0061 (15)	−0.0038 (16)
O2	0.0300 (19)	0.047 (2)	0.0323 (18)	0.0081 (16)	0.0003 (14)	−0.0103 (16)
O3	0.0242 (17)	0.045 (2)	0.0286 (17)	−0.0003 (15)	0.0019 (13)	−0.0086 (15)
O4	0.0203 (16)	0.0319 (17)	0.0329 (17)	0.0002 (13)	0.0004 (13)	−0.0083 (14)
O5	0.043 (2)	0.0330 (19)	0.0336 (18)	−0.0107 (16)	−0.0014 (15)	−0.0078 (15)
O6	0.0249 (17)	0.0347 (19)	0.0413 (19)	0.0031 (14)	0.0014 (14)	−0.0092 (15)
O7	0.169 (5)	0.051 (2)	0.073 (2)	−0.028 (2)	0.019 (3)	−0.0238 (19)
O8	0.169 (5)	0.051 (2)	0.073 (2)	−0.028 (2)	0.019 (3)	−0.0238 (19)
O9	0.063 (2)	0.080 (3)	0.137 (4)	0.007 (2)	0.051 (2)	−0.014 (3)
O10	0.063 (2)	0.080 (3)	0.137 (4)	0.007 (2)	0.051 (2)	−0.014 (3)

### Geometric parameters (Å, °)

**Table:**

C1—C4	1.580 (11)	C38—H38B	0.9600
C1—H1A	0.9600	C38—H38C	0.9600
C1—H1B	0.9600	C39—H39A	0.9600
C1—H1C	0.9600	C39—H39B	0.9600
C2—C4	1.475 (12)	C39—H39C	0.9600
C2—H2A	0.9600	C40—H40A	0.9600
C2—H2B	0.9600	C40—H40B	0.9600
C2—H2C	0.9600	C40—H40C	0.9600
C3—C4	1.550 (11)	C41—C42	1.532 (7)
C3—H3A	0.9600	C41—H41A	0.9700
C3—H3B	0.9600	C41—H41B	0.9700
C3—H3C	0.9600	C42—C47	1.384 (7)
C4—C5	1.548 (7)	C42—C43	1.404 (7)
C4—C2'	1.5498 (11)	C43—C44	1.409 (8)
C4—C1'	1.5501 (11)	C43—H43	0.9300
C4—C3'	1.5501 (11)	C44—C45	1.376 (8)
C5—C6	1.390 (8)	C44—C48	1.543 (7)
C5—C10	1.415 (8)	C45—C46	1.403 (7)
C6—C7	1.400 (7)	C45—H45	0.9300
C6—H6	0.9300	C46—C47	1.411 (7)
C7—C8	1.408 (7)	C46—C52	1.523 (7)
C7—C52	1.531 (7)	C47—O3	1.412 (5)
C8—O2	1.377 (5)	C48—C49	1.517 (10)
C8—C9	1.400 (7)	C48—C51	1.534 (9)
C9—C10	1.396 (7)	C48—C50	1.547 (9)
C9—C11	1.532 (7)	C49—H49A	0.9600
C10—H10	0.9300	C49—H49B	0.9600
C11—C12	1.524 (7)	C49—H49C	0.9600
C11—H11A	0.9700	C50—H50A	0.9600
C11—H11B	0.9700	C50—H50B	0.9600
C12—C17	1.367 (7)	C50—H50C	0.9600
C12—C13	1.422 (7)	C51—H51A	0.9600
C13—O4	1.386 (5)	C51—H51B	0.9600
C13—C14	1.407 (7)	C51—H51C	0.9600
C14—C15	1.388 (7)	C52—H52A	0.9700
C14—C30	1.532 (7)	C52—H52B	0.9700
C15—C16	1.400 (7)	C53—O3	1.438 (6)
C15—H15	0.9300	C53—C54	1.504 (7)
C16—C17	1.413 (7)	C53—H53A	0.9700
C16—C18	1.529 (7)	C53—H53B	0.9700
C17—H17	0.9300	C54—O6	1.431 (6)
C18—C21	1.524 (9)	C54—H54A	0.9700
C18—C19	1.544 (8)	C54—H54B	0.9700
C18—C20	1.567 (9)	C55—O6	1.372 (5)
C19—H19A	0.9600	C55—C60	1.382 (7)
C19—H19B	0.9600	C55—C56	1.387 (7)
C19—H19C	0.9600	C56—C57	1.392 (8)
C20—H20A	0.9600	C56—H56	0.9300
C20—H20B	0.9600	C57—C58	1.371 (8)
C20—H20C	0.9600	C57—H57	0.9300
C21—H21A	0.9600	C58—C59	1.371 (8)
C21—H21B	0.9600	C58—N1	1.467 (7)
C21—H21C	0.9600	C59—C60	1.391 (7)
C22—O4	1.443 (5)	C59—H59	0.9300
C22—C23	1.505 (7)	C60—H60	0.9300
C22—H22A	0.9700	C61—C62	1.444 (13)
C22—H22B	0.9700	C61—H61A	0.9600
C23—O5	1.434 (6)	C61—H61B	0.9600
C23—H23A	0.9700	C61—H61C	0.9600
C23—H23B	0.9700	C62—N3	1.133 (11)
C24—O5	1.360 (6)	C63—C64	1.436 (11)
C24—C29	1.385 (7)	C63—H63A	0.9600
C24—C25	1.405 (8)	C63—H63B	0.9600
C25—C26	1.378 (8)	C63—H63C	0.9600
C25—H25	0.9300	C64—N5	1.121 (9)
C26—C27	1.383 (8)	C65—C66	1.443 (9)
C26—H26	0.9300	C65—H65A	0.9600
C27—C28	1.383 (8)	C65—H65B	0.9600
C27—N2	1.463 (7)	C65—H65C	0.9600
C28—C29	1.378 (7)	C66—N4	1.131 (9)
C28—H28	0.9300	C67—N6	1.20 (3)
C29—H29	0.9300	C67—C68	1.529 (18)
C30—C31	1.542 (7)	C68—H68A	0.9600
C30—H30A	0.9700	C68—H68B	0.9600
C30—H30B	0.9700	C68—H68C	0.9600
C31—C36	1.377 (7)	C1'—H1'1	0.9600
C31—C32	1.417 (7)	C1'—H1'2	0.9600
C32—O1	1.369 (5)	C1'—H1'3	0.9600
C32—C33	1.396 (7)	C2'—H2'1	0.9600
C33—C34	1.390 (7)	C2'—H2'2	0.9600
C33—C41	1.545 (7)	C2'—H2'3	0.9600
C34—C35	1.390 (8)	C3'—H3'1	0.9600
C34—H34	0.9300	C3'—H3'2	0.9600
C35—C36	1.417 (7)	C3'—H3'3	0.9600
C35—C37	1.528 (7)	N1—O9	1.202 (7)
C36—H36	0.9300	N1—O10	1.210 (7)
C37—C38	1.534 (8)	N2—O7	1.212 (7)
C37—C39	1.540 (9)	N2—O8	1.222 (7)
C37—C40	1.546 (9)	O1—H1	0.8200
C38—H38A	0.9600	O2—H2	0.8200
			
C4—C1—H1A	109.5	H38A—C38—H38C	109.5
C4—C1—H1B	109.5	H38B—C38—H38C	109.5
H1A—C1—H1B	109.5	C37—C39—H39A	109.5
C4—C1—H1C	109.5	C37—C39—H39B	109.5
H1A—C1—H1C	109.5	H39A—C39—H39B	109.5
H1B—C1—H1C	109.5	C37—C39—H39C	109.5
C4—C2—H2A	109.5	H39A—C39—H39C	109.5
C4—C2—H2B	109.5	H39B—C39—H39C	109.5
H2A—C2—H2B	109.5	C37—C40—H40A	109.5
C4—C2—H2C	109.5	C37—C40—H40B	109.5
H2A—C2—H2C	109.5	H40A—C40—H40B	109.5
H2B—C2—H2C	109.5	C37—C40—H40C	109.5
C4—C3—H3A	109.5	H40A—C40—H40C	109.5
C4—C3—H3B	109.5	H40B—C40—H40C	109.5
H3A—C3—H3B	109.5	C42—C41—C33	111.6 (4)
C4—C3—H3C	109.5	C42—C41—H41A	109.3
H3A—C3—H3C	109.5	C33—C41—H41A	109.3
H3B—C3—H3C	109.5	C42—C41—H41B	109.3
C2—C4—C5	112.3 (6)	C33—C41—H41B	109.3
C2—C4—C3	111.4 (8)	H41A—C41—H41B	108.0
C5—C4—C3	107.2 (5)	C47—C42—C43	119.5 (5)
C2'—C4—C1'	119.7 (19)	C47—C42—C41	121.5 (4)
C2'—C4—C3'	109.6 (18)	C43—C42—C41	118.7 (5)
C1'—C4—C3'	99 (2)	C42—C43—C44	120.4 (5)
C2—C4—C1	112.0 (8)	C42—C43—H43	119.8
C5—C4—C1	111.0 (5)	C44—C43—H43	119.8
C3—C4—C1	102.3 (7)	C45—C44—C43	117.8 (4)
C6—C5—C10	117.0 (5)	C45—C44—C48	120.3 (5)
C6—C5—C4	122.9 (5)	C43—C44—C48	121.8 (5)
C10—C5—C4	120.1 (5)	C44—C45—C46	124.2 (5)
C5—C6—C7	122.9 (5)	C44—C45—H45	117.9
C5—C6—H6	118.6	C46—C45—H45	117.9
C7—C6—H6	118.6	C45—C46—C47	116.0 (5)
C6—C7—C8	118.5 (5)	C45—C46—C52	121.1 (5)
C6—C7—C52	119.7 (4)	C47—C46—C52	122.6 (4)
C8—C7—C52	121.6 (4)	C42—C47—C46	122.1 (4)
O2—C8—C9	122.2 (4)	C42—C47—O3	119.6 (4)
O2—C8—C7	117.2 (4)	C46—C47—O3	118.3 (4)
C9—C8—C7	120.6 (4)	C49—C48—C51	109.7 (6)
C10—C9—C8	119.0 (5)	C49—C48—C44	112.5 (5)
C10—C9—C11	118.9 (5)	C51—C48—C44	109.5 (5)
C8—C9—C11	121.9 (4)	C49—C48—C50	109.1 (6)
C9—C10—C5	122.1 (5)	C51—C48—C50	107.3 (6)
C9—C10—H10	118.9	C44—C48—C50	108.6 (5)
C5—C10—H10	118.9	C48—C49—H49A	109.5
C12—C11—C9	110.4 (4)	C48—C49—H49B	109.5
C12—C11—H11A	109.6	H49A—C49—H49B	109.5
C9—C11—H11A	109.6	C48—C49—H49C	109.5
C12—C11—H11B	109.6	H49A—C49—H49C	109.5
C9—C11—H11B	109.6	H49B—C49—H49C	109.5
H11A—C11—H11B	108.1	C48—C50—H50A	109.5
C17—C12—C13	119.6 (4)	C48—C50—H50B	109.5
C17—C12—C11	118.9 (5)	H50A—C50—H50B	109.5
C13—C12—C11	121.2 (4)	C48—C50—H50C	109.5
O4—C13—C14	122.8 (4)	H50A—C50—H50C	109.5
O4—C13—C12	116.9 (4)	H50B—C50—H50C	109.5
C14—C13—C12	120.3 (4)	C48—C51—H51A	109.5
C15—C14—C13	117.5 (4)	C48—C51—H51B	109.5
C15—C14—C30	118.2 (4)	H51A—C51—H51B	109.5
C13—C14—C30	124.0 (4)	C48—C51—H51C	109.5
C14—C15—C16	123.9 (5)	H51A—C51—H51C	109.5
C14—C15—H15	118.0	H51B—C51—H51C	109.5
C16—C15—H15	118.0	C46—C52—C7	111.0 (4)
C15—C16—C17	116.4 (4)	C46—C52—H52A	109.4
C15—C16—C18	124.0 (5)	C7—C52—H52A	109.4
C17—C16—C18	119.5 (5)	C46—C52—H52B	109.4
C12—C17—C16	122.1 (5)	C7—C52—H52B	109.4
C12—C17—H17	118.9	H52A—C52—H52B	108.0
C16—C17—H17	118.9	O3—C53—C54	106.3 (4)
C21—C18—C16	112.0 (5)	O3—C53—H53A	110.5
C21—C18—C19	109.1 (5)	C54—C53—H53A	110.5
C16—C18—C19	109.3 (4)	O3—C53—H53B	110.5
C21—C18—C20	108.3 (5)	C54—C53—H53B	110.5
C16—C18—C20	109.2 (4)	H53A—C53—H53B	108.7
C19—C18—C20	109.0 (5)	O6—C54—C53	106.7 (4)
C18—C19—H19A	109.5	O6—C54—H54A	110.4
C18—C19—H19B	109.5	C53—C54—H54A	110.4
H19A—C19—H19B	109.5	O6—C54—H54B	110.4
C18—C19—H19C	109.5	C53—C54—H54B	110.4
H19A—C19—H19C	109.5	H54A—C54—H54B	108.6
H19B—C19—H19C	109.5	O6—C55—C60	116.3 (4)
C18—C20—H20A	109.5	O6—C55—C56	123.3 (4)
C18—C20—H20B	109.5	C60—C55—C56	120.4 (4)
H20A—C20—H20B	109.5	C55—C56—C57	119.0 (5)
C18—C20—H20C	109.5	C55—C56—H56	120.5
H20A—C20—H20C	109.5	C57—C56—H56	120.5
H20B—C20—H20C	109.5	C58—C57—C56	120.0 (5)
C18—C21—H21A	109.5	C58—C57—H57	120.0
C18—C21—H21B	109.5	C56—C57—H57	120.0
H21A—C21—H21B	109.5	C57—C58—C59	121.5 (5)
C18—C21—H21C	109.5	C57—C58—N1	119.5 (5)
H21A—C21—H21C	109.5	C59—C58—N1	118.9 (5)
H21B—C21—H21C	109.5	C58—C59—C60	118.9 (5)
O4—C22—C23	109.3 (4)	C58—C59—H59	120.6
O4—C22—H22A	109.8	C60—C59—H59	120.6
C23—C22—H22A	109.8	C55—C60—C59	120.2 (5)
O4—C22—H22B	109.8	C55—C60—H60	119.9
C23—C22—H22B	109.8	C59—C60—H60	119.9
H22A—C22—H22B	108.3	C62—C61—H61A	109.5
O5—C23—C22	108.5 (4)	C62—C61—H61B	109.5
O5—C23—H23A	110.0	H61A—C61—H61B	109.5
C22—C23—H23A	110.0	C62—C61—H61C	109.5
O5—C23—H23B	110.0	H61A—C61—H61C	109.5
C22—C23—H23B	110.0	H61B—C61—H61C	109.5
H23A—C23—H23B	108.4	N3—C62—C61	179.9 (13)
O5—C24—C29	124.8 (5)	C64—C63—H63A	109.5
O5—C24—C25	116.2 (4)	C64—C63—H63B	109.5
C29—C24—C25	119.0 (5)	H63A—C63—H63B	109.5
C26—C25—C24	121.3 (5)	C64—C63—H63C	109.5
C26—C25—H25	119.3	H63A—C63—H63C	109.5
C24—C25—H25	119.3	H63B—C63—H63C	109.5
C25—C26—C27	118.7 (6)	N5—C64—C63	178.7 (9)
C25—C26—H26	120.7	C66—C65—H65A	109.5
C27—C26—H26	120.7	C66—C65—H65B	109.5
C28—C27—C26	120.5 (5)	H65A—C65—H65B	109.5
C28—C27—N2	120.2 (5)	C66—C65—H65C	109.5
C26—C27—N2	119.4 (5)	H65A—C65—H65C	109.5
C29—C28—C27	121.0 (5)	H65B—C65—H65C	109.5
C29—C28—H28	119.5	N4—C66—C65	179.3 (8)
C27—C28—H28	119.5	N6—C67—C68	165 (3)
C28—C29—C24	119.5 (5)	C67—C68—H68A	109.5
C28—C29—H29	120.2	C67—C68—H68B	109.5
C24—C29—H29	120.2	H68A—C68—H68B	109.5
C14—C30—C31	111.1 (4)	C67—C68—H68C	109.5
C14—C30—H30A	109.4	H68A—C68—H68C	109.5
C31—C30—H30A	109.4	H68B—C68—H68C	109.5
C14—C30—H30B	109.4	C4—C1'—H1'1	109.5
C31—C30—H30B	109.4	C4—C1'—H1'2	109.5
H30A—C30—H30B	108.0	H1'1—C1'—H1'2	109.5
C36—C31—C32	118.1 (4)	C4—C1'—H1'3	109.5
C36—C31—C30	121.3 (4)	H1'1—C1'—H1'3	109.5
C32—C31—C30	120.4 (4)	H1'2—C1'—H1'3	109.5
O1—C32—C33	124.6 (4)	C4—C2'—H2'1	109.5
O1—C32—C31	115.0 (4)	C4—C2'—H2'2	109.5
C33—C32—C31	120.4 (4)	H2'1—C2'—H2'2	109.5
C34—C33—C32	119.5 (5)	C4—C2'—H2'3	109.5
C34—C33—C41	118.5 (4)	H2'1—C2'—H2'3	109.5
C32—C33—C41	121.9 (4)	H2'2—C2'—H2'3	109.5
C35—C34—C33	121.9 (5)	C4—C3'—H3'1	109.5
C35—C34—H34	119.1	C4—C3'—H3'2	109.5
C33—C34—H34	119.1	H3'1—C3'—H3'2	109.5
C34—C35—C36	117.2 (5)	C4—C3'—H3'3	109.5
C34—C35—C37	123.2 (5)	H3'1—C3'—H3'3	109.5
C36—C35—C37	119.6 (5)	H3'2—C3'—H3'3	109.5
C31—C36—C35	122.8 (5)	O9—N1—O10	120.8 (5)
C31—C36—H36	118.6	O9—N1—C58	120.6 (5)
C35—C36—H36	118.6	O10—N1—C58	118.5 (5)
C35—C37—C38	109.5 (4)	O7—N2—O8	121.7 (6)
C35—C37—C39	109.6 (5)	O7—N2—C27	118.6 (5)
C38—C37—C39	108.9 (5)	O8—N2—C27	119.7 (5)
C35—C37—C40	112.1 (5)	C32—O1—H1	109.5
C38—C37—C40	108.4 (5)	C8—O2—H2	109.5
C39—C37—C40	108.2 (5)	C47—O3—C53	113.8 (4)
C37—C38—H38A	109.5	C13—O4—C22	117.7 (3)
C37—C38—H38B	109.5	C24—O5—C23	119.7 (4)
H38A—C38—H38B	109.5	C55—O6—C54	119.8 (4)
C37—C38—H38C	109.5		
			
C2—C4—C5—C6	−123.5 (9)	C31—C32—C33—C41	178.4 (4)
C3—C4—C5—C6	113.8 (7)	C32—C33—C34—C35	−1.5 (7)
C2'—C4—C5—C6	59.9 (16)	C41—C33—C34—C35	−178.0 (5)
C1'—C4—C5—C6	−164.7 (18)	C33—C34—C35—C36	−0.4 (7)
C3'—C4—C5—C6	−57.7 (14)	C33—C34—C35—C37	179.1 (5)
C1—C4—C5—C6	2.8 (8)	C32—C31—C36—C35	−1.3 (7)
C2—C4—C5—C10	58.5 (9)	C30—C31—C36—C35	173.5 (4)
C3—C4—C5—C10	−64.2 (8)	C34—C35—C36—C31	1.9 (7)
C2'—C4—C5—C10	−118.1 (15)	C37—C35—C36—C31	−177.7 (4)
C1'—C4—C5—C10	17.3 (19)	C34—C35—C37—C38	116.4 (6)
C3'—C4—C5—C10	124.2 (13)	C36—C35—C37—C38	−64.1 (7)
C1—C4—C5—C10	−175.2 (6)	C34—C35—C37—C39	−124.1 (6)
C10—C5—C6—C7	0.8 (8)	C36—C35—C37—C39	55.4 (7)
C4—C5—C6—C7	−177.3 (5)	C34—C35—C37—C40	−3.9 (8)
C5—C6—C7—C8	−0.7 (7)	C36—C35—C37—C40	175.6 (5)
C5—C6—C7—C52	174.2 (5)	C34—C33—C41—C42	90.1 (5)
C6—C7—C8—O2	−177.2 (4)	C32—C33—C41—C42	−86.3 (5)
C52—C7—C8—O2	8.0 (7)	C33—C41—C42—C47	91.5 (5)
C6—C7—C8—C9	0.0 (7)	C33—C41—C42—C43	−82.6 (5)
C52—C7—C8—C9	−174.8 (4)	C47—C42—C43—C44	0.3 (7)
O2—C8—C9—C10	177.6 (4)	C41—C42—C43—C44	174.6 (4)
C7—C8—C9—C10	0.5 (7)	C42—C43—C44—C45	−1.9 (7)
O2—C8—C9—C11	−7.8 (7)	C42—C43—C44—C48	178.8 (5)
C7—C8—C9—C11	175.1 (4)	C43—C44—C45—C46	1.8 (8)
C8—C9—C10—C5	−0.3 (7)	C48—C44—C45—C46	−178.9 (5)
C11—C9—C10—C5	−175.1 (4)	C44—C45—C46—C47	−0.1 (7)
C6—C5—C10—C9	−0.3 (7)	C44—C45—C46—C52	−173.2 (4)
C4—C5—C10—C9	177.9 (5)	C43—C42—C47—C46	1.4 (7)
C10—C9—C11—C12	86.4 (5)	C41—C42—C47—C46	−172.7 (4)
C8—C9—C11—C12	−88.2 (5)	C43—C42—C47—O3	179.2 (4)
C9—C11—C12—C17	−82.2 (5)	C41—C42—C47—O3	5.1 (7)
C9—C11—C12—C13	90.9 (5)	C45—C46—C47—C42	−1.5 (7)
C17—C12—C13—O4	178.8 (4)	C52—C46—C47—C42	171.5 (4)
C11—C12—C13—O4	5.7 (6)	C45—C46—C47—O3	−179.3 (4)
C17—C12—C13—C14	0.0 (7)	C52—C46—C47—O3	−6.3 (7)
C11—C12—C13—C14	−173.0 (4)	C45—C44—C48—C49	−173.3 (6)
O4—C13—C14—C15	−179.0 (4)	C43—C44—C48—C49	6.0 (8)
C12—C13—C14—C15	−0.4 (6)	C45—C44—C48—C51	−51.1 (7)
O4—C13—C14—C30	−5.2 (7)	C43—C44—C48—C51	128.2 (6)
C12—C13—C14—C30	173.5 (4)	C45—C44—C48—C50	65.8 (7)
C13—C14—C15—C16	−1.0 (7)	C43—C44—C48—C50	−114.9 (6)
C30—C14—C15—C16	−175.2 (4)	C45—C46—C52—C7	74.4 (5)
C14—C15—C16—C17	2.5 (7)	C47—C46—C52—C7	−98.3 (5)
C14—C15—C16—C18	−179.6 (4)	C6—C7—C52—C46	−78.2 (5)
C13—C12—C17—C16	1.6 (7)	C8—C7—C52—C46	96.5 (5)
C11—C12—C17—C16	174.9 (4)	O3—C53—C54—O6	62.0 (6)
C15—C16—C17—C12	−2.8 (7)	O6—C55—C56—C57	−177.3 (6)
C18—C16—C17—C12	179.2 (4)	C60—C55—C56—C57	1.6 (9)
C15—C16—C18—C21	5.0 (8)	C55—C56—C57—C58	−0.7 (10)
C17—C16—C18—C21	−177.2 (5)	C56—C57—C58—C59	0.0 (11)
C15—C16—C18—C19	−116.0 (6)	C56—C57—C58—N1	177.4 (6)
C17—C16—C18—C19	61.9 (7)	C57—C58—C59—C60	−0.2 (9)
C15—C16—C18—C20	124.9 (5)	N1—C58—C59—C60	−177.6 (6)
C17—C16—C18—C20	−57.3 (6)	O6—C55—C60—C59	177.2 (5)
O4—C22—C23—O5	65.7 (5)	C56—C55—C60—C59	−1.8 (8)
O5—C24—C25—C26	−179.5 (5)	C58—C59—C60—C55	1.1 (8)
C29—C24—C25—C26	−0.8 (9)	C57—C58—N1—O9	−173.1 (7)
C24—C25—C26—C27	−0.2 (10)	C59—C58—N1—O9	4.3 (10)
C25—C26—C27—C28	−0.1 (9)	C57—C58—N1—O10	9.2 (10)
C25—C26—C27—N2	−178.8 (6)	C59—C58—N1—O10	−173.4 (7)
C26—C27—C28—C29	1.4 (8)	C28—C27—N2—O7	−9.7 (9)
N2—C27—C28—C29	−179.9 (5)	C26—C27—N2—O7	169.0 (7)
C27—C28—C29—C24	−2.5 (7)	C28—C27—N2—O8	173.7 (6)
O5—C24—C29—C28	−179.3 (5)	C26—C27—N2—O8	−7.6 (9)
C25—C24—C29—C28	2.1 (7)	C42—C47—O3—C53	90.6 (5)
C15—C14—C30—C31	74.8 (5)	C46—C47—O3—C53	−91.6 (5)
C13—C14—C30—C31	−99.0 (5)	C54—C53—O3—C47	−178.3 (4)
C14—C30—C31—C36	−85.5 (5)	C14—C13—O4—C22	−62.0 (6)
C14—C30—C31—C32	89.2 (5)	C12—C13—O4—C22	119.3 (4)
C36—C31—C32—O1	−179.7 (4)	C23—C22—O4—C13	−133.4 (4)
C30—C31—C32—O1	5.5 (6)	C29—C24—O5—C23	−9.1 (7)
C36—C31—C32—C33	−0.7 (7)	C25—C24—O5—C23	169.5 (5)
C30—C31—C32—C33	−175.6 (4)	C22—C23—O5—C24	180.0 (4)
O1—C32—C33—C34	−179.1 (4)	C60—C55—O6—C54	166.9 (4)
C31—C32—C33—C34	2.1 (7)	C56—C55—O6—C54	−14.2 (7)
O1—C32—C33—C41	−2.7 (7)	C53—C54—O6—C55	−177.4 (4)

### Hydrogen-bond geometry (Å, °)

**Table:**

*D*—H···*A*	*D*—H	H···*A*	*D*···*A*	*D*—H···*A*
O1—H1···O3	0.82	1.99	2.802 (6)	171
O2—H2···O4	0.82	1.97	2.781 (4)	176
C50—H50C···O7^i^	0.96	2.59	3.487 (10)	156
C68—H68B···O10	0.96	2.39	3.247 (13)	148
C65—H65A···Cg1	0.96	2.66	3.590 (6)	163

Symmetry codes: (i) −*x*+1, −*y*+1, −*z*+1.
